# A De Novo *POLD1* Mutation Associated With Mandibular Hypoplasia, Deafness, Progeroid Features, and Lipodystrophy Syndrome in a Family With Werner Syndrome

**DOI:** 10.1177/2324709618786770

**Published:** 2018-07-12

**Authors:** Linda R. Wang, Aleksandar Radonjic, Allison A. Dilliott, Adam D. McIntyre, Robert A. Hegele

**Affiliations:** 1 Western University, London, Ontario, Canada

**Keywords:** aging, progeria, whole exome sequencing, diabetes, lipodystrophy, monogenic

## Abstract

*Background.* Mandibular hypoplasia, deafness, progeroid features, and lipodystrophy (MDPL) syndrome is a recently recognized genetic disorder comprised of mandibular hypoplasia, deafness, progeroid features, and lipodystrophy. It is caused by an autosomal dominant mutation in the *POLD1* gene, with <20 genetically confirmed cases to date. Clinical overlap with other progeroid syndromes including Werner syndrome (WS) can present diagnostic challenges. *Case.* The proband is a 36-year-old male of Sicilian ancestry who was phenotypically normal at birth. Onset of lipodystrophic and progeroid features began at 18 months, with progressive loss of subcutaneous fat, prominent eyes, and pinched nose. Over the next 2 decades, he developed hearing loss, small fingers, joint contractures, hypogonadism, osteoporosis, and hypertriglyceridemia. Three of his 4 siblings had premature hair graying and loss, severe bilateral cataracts, skin changes, and varying degrees of age-related metabolic conditions, raising suspicion for a genetic progeroid syndrome. *Genetic Analysis.* A targeted sequencing panel identified a heterozygous *WRN* mutation in the proband’s genomic DNA. Sanger sequencing further revealed his parents and an asymptomatic brother to be carriers of this mutation, and in his 3 brothers affected with classic WS the mutation was identified in the homozygous state. Whole exome sequencing ultimately revealed the proband harbored the causative de novo in-frame deletion in *POLD1* (p.Ser605del), which is the most common mutation in MDPL patients. *Conclusion.* We report the unusual convergence of 2 rare progeroid disorders in the same family: the proband displayed sporadic MDPL syndrome, while 3 brothers had classical autosomal recessive WS. Whole exome sequencing was invaluable in clarifying the molecular diagnoses in this family.

## Introduction

Progeroid syndromes comprise a group of heterogeneous disorders characterized by features of accelerated aging with multisystem involvement. Werner syndrome (WS; MIM #277700), sometimes termed progeria of adulthood, is a rare autosomal recessive disorder caused by a defective helicase due to homozygous or compound heterozygous mutations in the WS RecQ-like Helicase gene (*WRN*; MIM #604611). Typical features include bilateral cataracts, hair graying or thinning, sclerodermatous skin with wizened facial features, thin limbs and stocky trunk, and type 2 diabetes mellitus (T2DM).

Mandibular hypoplasia, deafness, and progeroid features with lipodystrophy (MDPL; MIM #615381) syndrome is a recently discovered, exceedingly rare autosomal dominant disorder caused by mutations in the DNA polymerase delta 1, catalytic subunit gene (*POLD1*), encoding a catalytic subunit of the lagging strand DNA polymerase, resulting in genomic instability, and cellular senescence. Since its first description in 2010^1^ and mechanistic explanation in 2013,^2^ 22 cases have been reported worldwide, 15 of which have been molecularly characterized. A de novo in-frame deletion of a highly conserved serine residue in *POLD1*, namely, p.Ser605del, is the most frequently implicated causative variant.^3^

We describe a unique family with phenotypically normal parents and 4 children with progeroid features, resulting from 2 distinct etiologies: WS and MDPL syndrome. This is the 16th molecularly characterized case of MDPL syndrome.

## Subjects and Methods

### Proband and Family Members

**Figure 1.**
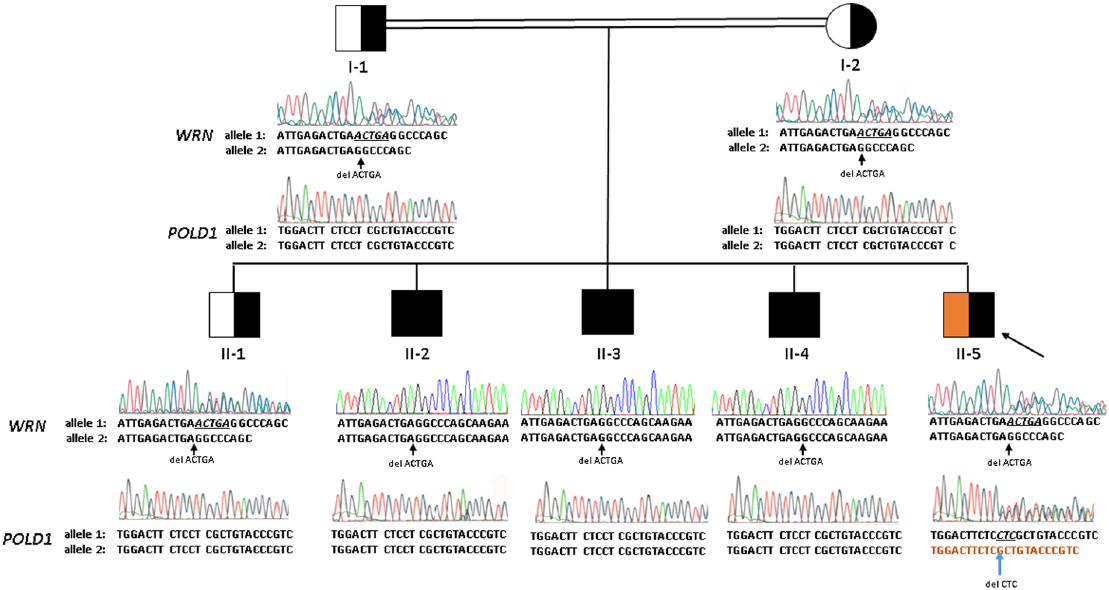
Pedigree showing proband (II-5, large arrow) in relation to parents (I-1, I-2) and 4 brothers (II-1 through II-4). Males and females are squares and circles, respectively. Consanguinity is indicated by double lines. Black shading indicates the genotype status for the *WRN* variant (half- and whole-filled symbols are heterozygotes and homozygotes, respectively). Orange shading indicates the genotype status for the *POLD1* variant (half-filled symbol is a heterozygote). Below each individual, Sanger sequence electropherogram tracings for *WRN* and *POLD1* are shown; nucleotide calls for each allele are shown beneath the sequence tracings. Individuals I-1, I-2, II-1, and II-5 each have a heterozygous genotype for *WRN* p.Thr307Thr fsX5, consisting of one wild-type allele with normal sequence and one frameshifted allele due to the deletion of 5 base pairs, indicated by small arrows and reflected in the reduced intensity and mixed colors of peaks following the deletion. Individuals II-2, II-3, and II-4 are homozygous for this mutation. Similarly, the tracings for the affected region of the *POLD1* gene are shown; all individuals except for II-5 are homozygous for the normal wild-type sequence, while individual II-5 has a de novo heterozygous in-frame deletion of 3 nucleotides, involving codon 605; the mutation is designated as *POLD1* p.Ser605del and its position is indicated by the small arrow.

The proband (Figure 1; subject II-5) is a 36-year-old man who presented with long-standing lipodystrophy and progeroid features within an unusual family. He is the youngest of 5 sons, born in Canada to Sicilian parents (Figure 1; subjects I-1 and I-2) who shared the same great-grandparents. His family history was unremarkable.

His eldest brother (Figure 1; II-1) had normal development and phenotypic features. His other 3 brothers (Figure 1; subjects II-2, II-3, and II-4) all displayed features of premature aging in the third to fourth decades of life, including bilateral cataracts, premature hair graying and loss, premature onset of T2DM, hyperlipidemia, osteoporosis, and/or chronic skin ulcers.

The proband’s clinical trajectory departed dramatically from those of his brothers. He had normal length and weighed 9 pounds at birth. At around 18 months of age, he developed progressive dysmorphic features, disappearance of subcutaneous fat, and failure to thrive. Despite good appetite, extensive investigation excluding endocrinological, genetic, malabsorptive, and systemic disorders, as well as a trial of growth hormone and testosterone, he remained very thin. Other striking features included prominent eyes, high-pitched voice, thin limbs, thin and tight skin complicated by tearing, bullae, and recurrent cellulitis. His medical history includes sensorineural hearing loss requiring hearing aids at age 10 years, elbow contractures occasionally complicated by ulceration, hypogonadism, osteoporosis, and hypertriglyceridemia. His medications include fenofibrate, rosuvastatin, vitamin D_2_, fish oil, coenzyme Q10, and vitamin C. He had no features suggestive of cardiac, peripheral vascular, pancreatobiliary, or renal disease, and no smoking or alcohol use.

Physical examination revealed progeroid morphology with narrowed nose and ears, thin skin, and hollowed cheeks. His weight and height were 39.5 kg and 1.62 m, respectively, with body mass index of 15.6 kg/m^2^. Blood pressure was 109/75 mm Hg, and radial pulse was regular at 92 beats per minute. The near absence of subcutaneous tissue from his limbs resulted in an almost translucent skin quality. His hands were small, with very fine bones and tight skin over the fingers. Heart and lung sounds were normal. His abdomen was firm with no organomegaly. He had brisk patellar reflex bilaterally. Fundoscopy was normal, and no cataracts were detected. Investigations and clinical data from patient and family members are presented in Table 1.

**Table 1.** Phenotypic Features of the Proband and Nuclear Family^a^.

Features	I-1	I-2	II-1	II-2	II-3	II-4	II-5	WS	MDPL
**Age**	71	61	44	43	41	39	36		
**Height (m)**	1.6	1.52	1.63	1.62	1.57	1.64	1.62		
**Weight (kg)**	77.8	76.7	93.5	65.0	61.4	79.6	39.5		
**BMI (kg/m** ^2^ **)**	30.4	33.2	35.2	24.8	24.9	29.6	15.1		
**Bilateral cataracts (age at surgery)**	N	N	N	Y (32)	Y (36)	Y (32)	N	Y	N
**Hearing impairment (age)**	N	N	N	N	N	N	Y (10)	N	Y
**Joint contractures**	N	N	N	N	N	N	Y (elbows)	N	Y
**Dental crowding**	N	N	N	N	N	N	Y	N	Y
**Tight skin around cheeks**	N	N	N	—	—	—	Y	Y	Y
**Premature hair graying**	N	N	Y	Y	Y	Y	N	Y	Y
**Mandibular hypoplasia**	N	N	N	—	N	N	Y	Y	Y
**High-pitched voice**	N	N	N	N	N	N	Y	Y	Y
**Short stature**	Y	Y	Y	Y	Y	Y	Y	Y	Y
**Muscle wasting**	N	N	N	—	—	—	Y	Y	Y
**Loss of subcutaneous fat**	N	N	N	—	N	Y (feet)	Y	Y	Y
**Dyslipidemia**	Y	N	Y	Y	N	Y	Y	Y	Y
**Osteoporosis (age)**	N	N	N	—	Y (34)	—	Y (9)	Y	Y
**T2DM**	N	N	N	Y	—	Y	N	Y	Y
**Hypogonadism**	N	N	Y	—	N	Y	Y	Y	Y
**Thin limbs/wide trunk**	N	N	N	Y	—	—	Y	Y	Y
**Neoplasms**	N	N	N	N	N	N	N	Y	N
**Skin atrophy**	N	N	N	?	N	Y^b^	N	Y	N
**Angina or claudication**	N	N	N	N	N	N	N	Y	—
**Ulcerations**	N	N	N	Y^b^	Y (elbow)	Y (elbow)	Y (elbow)	Y	N
**Vascular insufficiency**	N	N	N	Y	N	N	N	Y	N
**Peripheral neuropathy**	N	N	N	Y (motor)	N	Y (sensory)	N	Y	N
**Hypothyroidism**	N	N	N	Y	Y	N	N	—	—
**Cognition**	Normal	Normal	Normal	Normal	Normal	Normal	Normal	Normal	Normal
**HbA1c**	5.8%		6.0%	7.7%		6.9%	5.4%		
**Hepatic steatosis on US**	—	Y	—	—	—	—	Y		
**ALT (<41 U/L)**	17	—	36	—		46			
**AST (<45 U/L)**	74	15	—	20	—	17	17		
**ALP (40-129 U/L)**	—	—	76	—		—			
**Total C (mmol/L)**	6.29	5.25	5.98	6.55	—	5.45	7.3		
**LDL-C (mmol/L)**	4.2	3.1	4.09	4.57	—	2.93	—		
**HDL-C (mmol/L)**	1.06	1.91	0.98	1.32	—	0.66	0.78		
**TG (mmol/L)**	2.27	0.52	2.0	1.45	—	4.09	8.87		
**Mean carotid IMT (mm)**	—	0.69	0.65	0.94	—	0.5	0.53		
**Carotid IMT percentile**	90	50	60 (50-75)	>95	—	15 (10-25)	30 (25-50)		
**Apo A1 (1.2-1.4 g/L)**	1.16	1.51	1.23	1.38		1.17	1.93		
**Apo B (0.8-1.2 g/L)**	1.32	0.94		1.39					
**Apo B:A1 ratio**	1.14	0.62		1.01					
**Comments**			OSA	WPW, bursitis, severe heel ulcers, OM requiring debridement	MVC, TBI, migraine with aura	Skin atrophy (elbows, knuckles, plantar)	Skin tears, cellulitis, plantar calluses, dysphagia, fragility fractures, lagophthalmos		

Abbreviations: WS, Werner syndrome; MDPL, mandibular hypoplasia, deafness, progeroid features and lipodystrophy syndrome; N, feature absent; Y, yes/feature present; BMI, body mass index; T2DM, type 2 diabetes mellitus; HbA1c, hemoglobin A1c; US, ultrasound; ALT, alanine aminotransferase; AST, aspartate aminotransferase; ALP, alkaline phosphatase; total C, total cholesterol; LDL-C, low-density lipoprotein cholesterol; HDL, high-density lipoprotein cholesterol; TG, triglycerides; Apo, apolipoprotein; OSA, obstructive sleep apnea; WPW, Wolff-Parkinson White syndrome; OM, osteomyelitis; MVC, motor vehicle collision; TBI, traumatic brain injury.

a Typical MDPL features are in orange, WS features in green, and blue indicates overlapping features. Balding is usually present in WS but absent in MDPL. Normal ranges for some biochemical variables are shown. “—” represents unavailable or unknown information.

b Indicates further details in comments.

### DNA Sequencing and Genetic Analysis

Genomic DNA samples were isolated from the peripheral blood cells of each family member using the Gentra Puregene Blood kit (Qiagen, Venlo, The Netherlands). The proband’s DNA sample was subjected to targeted next-generation sequencing of all known lipodystrophy genes, including *LMNA, WRN, ZMPSTE24, PPARG*, and *EMD*, using LipidSeq on the MiSeq personal sequencer (Illumina, San Diego, CA), as previously described.^4-6^ Variant annotation and prioritization software VarSeq (Golden Helix, Bozeman, MT) was used to prioritize rare variants of nonsynonymous sequence ontology, likely to be contributing to disease presentation. Predictive algorithms for pathogenicity, such as Combined Annotation Dependent Depletion (CADD^7^; http://cadd.gs.washington.edu/); Sorting Intolerant From Tolerant (SIFT^8^; http://sift.jcvi.org/); and Polymorphism Phenotyping tool version 2 (PolyPhen-2^9^; http://genetics.bwh.harvard.edu/pph2/) were used to prioritize likely pathogenic variants. Minor allele frequency of each variant were determined based on those cited for Caucasian subpopulations in the Exome Aggregation Consortium database (ExAC^10^; http://exac.broadinstitute.org/); 1000 Genomes database (1000G^11^; http://internationalgenome.org/); and the National Heart, Lung, and Blood Institute Exome Sequencing Project (ESP; http://evs.gs.washington.edu/EVS/). All variants of likely clinical significance were confirmed in the proband and screened for in the other family members using Sanger sequencing.

Further investigation included whole exome sequencing (WES) performed on the genomic DNA of the proband. TruSeq Rapid Exome chemistry (Illumina, San Diego, CA) was used to create the sequence library. The library was sequenced, multiplexed with 5 other samples from unrelated patients submitted for WES, on a NextSeq 500 sequencing system (Illumina, San Diego, CA) using a 300-cycle high-output cartridge run at 2 × 150 paired-end sequencing mode. VarSeq was used to prioritize rare variants likely to be of clinical significance. Variants were screened for those that were nonsynonymous in sequence ontology, resulting in missense, insertion/deletion, splicing, or nonsense variation. Based on the family history of the proband, the inheritance pattern was suspected to be de novo autosomal dominant, meaning variants of heterozygous zygosity were prioritized. Variants were also prioritized if they were not present in the population databases ExAC, 1000G, and ESP. Remaining variants were then manually curated using the predictive algorithms for pathogenicity, the Online Mendelian Inheritance in Man database (https://omim.org/), and literature review to identify that which was of clinical significance. The identified mutation was confirmed by Sanger sequencing and an updated version of the LipidSeq panel, which contained the gene in which the mutation was identified. All other family members were screened for this mutation by Sanger sequencing.

## Results

Targeted next-generation sequencing revealed a heterozygous frameshift mutation in *WRN* (c.919_923delACTGA, p.Thr307ThrfsX5) in the proband (II-5). The heterozygous mutation was also identified in both parents and the eldest son (II-1), who are phenotypically normal, using Sanger sequencing, despite the proband (II-5) having the most dramatic clinical presentation. However, the mutation was identified in the homozygous state in subjects II-2, II-3, and II-4 by Sanger sequencing, in keeping with phenotype segregation for clinical WS (Figure 1). The mutation in *WRN* was determined to be novel according to our best knowledge and was absent from the ExAC, 1000G, and ESP databases. Predictive algorithms for pathogenicity SIFT and PolyPhen-2 did not cover this mutation; however, CADD strongly predicted the mutation to be pathogenic (CADD Phred score = 35.0). The mutation was confirmed with Sanger sequencing in the proband.

WES of the proband’s genomic DNA was undertaken to identify other rare and likely deleterious variants in functional candidate genes, using our established protocols and bioinformatics pipeline.^12-14^ In total, 36 171 variants were identified in the proband’s exome. Upon prioritization of variants nonsynonymous in sequence ontology, of heterozygous zygosity, and absent from population databases ExAC, 1000G, and ESP, 575 candidate variants remained. These candidate variants were manually curated using predictive algorithms for pathogenicity, Online Mendelian Inheritance in Man database, and literature review. After this review one candidate variant was identified: a heterozygous in-frame deletion in *POLD1* (c.1812_1814delCTC, p.Ser605del), already known to cause most cases of MDPL.^3^ CADD predicted the mutation is pathogenic (CADD Phred score = 22.3). The mutation was confirmed with both Sanger sequencing and an updated version of the targeted panel, LipidSeq, containing the *POLD1* gene. Sanger sequencing of all other family members was negative for the variant, confirming a de novo mutation.

## Discussion

We report a remarkable family at the intersection of multiple ultra-rare genetic events, resulting in 2 unrelated progeroid syndromes in a single generation. Both asymptomatic parents were carriers of the ultra-rare autosomal recessive *WRN* mutation, and 3 of their 5 children inherited homozygous alleles resulting in clinical WS. The proband, who was only a *WRN* carrier, harbored an even rarer de novo mutation (*POLD1* p.Ser605del) causing classic features of MDPL syndrome, which was identified using WES. We are not aware of any single kindred in which 2 unrelated rare progeroid syndromes have affected multiple members of the same generation.

Until recently, the conditions clinically overlapping with WS included mainly Hutchinson-Gilford progeria syndrome, mandibuloacral dysplasia, and atypical WS.^15-18^ In 2010, Shastry et al^1^ described 7 patients with a unique constellation of features distinct from these progeroid entities, namely, mandibular hypoplasia, hearing loss, progeroid features, lipodystrophy, and hypogonadism in males. Affected subjects lacked the clavicular hypoplasia or alopecia characteristic of mandibuloacral dysplasia, cataracts or neoplasms characteristic of WS, or known mutations for progeroid syndromes.

Within 3 years, Weedon et al identified the cause: a heterozygous in-frame deletion of a highly conserved serine residue (c.1812_1814delCTC, p.Ser605del) in the *POLD1* gene on 19q13.33.^2^ *POLD1* encodes the catalytic subunit of the key lagging-strand polymerase, a fundamental component of successful DNA replication. Decoupling of enzymatic activities were demonstrated in the *POLD1* p.Ser605del mutant, suggesting that an inability to efficiently incorporate dNTPs was to blame for genomic instability and cellular senescence.

To date, 22 MDPL patients have been reported, of whom 15 were characterized at the molecular level. A variety of ethnic backgrounds have been reported, and all were de novo mutations. *POLD1* p.Ser605del remains the most reported causal variant, although various missense mutations have also been found.^3^

The unique coincidence of WS and MDPL in a single family allows for nuanced comparison. The brothers with WS (II-2, II-3, II-4) all displayed its cardinal features: premature bilateral cataracts (present in virtually all WS patients), premature hair graying or thinning, short stature, and scleroderma-like skin resulting in pinched facial features beginning in the second decade. Other typical features varied among the siblings, including slender limbs, stocky trunk, voice changes, hypogonadism, soft tissue calcification, osteoporosis in the long bones, T2DM, severe atherosclerosis, and chronic ankle ulcers, consistent with the variable clinical spectrum in WS.^19,20^ The increased prevalence of unusual malignancies in WS, particularly sarcomas, meningiomas, thyroid neoplasms, and melanomas, have so far not been observed in the affected individuals in this family, who are in their fourth to fifth decades of life.

The proband exhibited classic MDPL features, several of which overlap with WS, including characteristic facial features, body habitus, insulin resistance, T2DM, hypertriglyceridemia, and hepatic steatosis.^3^ However, several key features help to distinguish the 2 conditions. Both are phenotypically normal in infancy; however, progressive lipodystrophy, characteristic facial features, and high-pitched voice begin in early childhood in MDPL, whereas the onset of these features before adolescence excludes the diagnosis of WS. Hearing loss is classically thought to be an obligate feature of MDPL, although rare exceptions have recently been found.^21^ Cataracts are not typical of MDPL. Hypogonadism and cryptorchidism are common among MDPL males, and prominent eyes and dental crowding are particularly pronounced in MDPL. Although much remains to be learned about the natural history of MDPL, existing data suggest potentially longer lifespan than WS, and have not indicated increased malignancy risk as seen in WS. It will be interesting to observe whether this holds true for this MDPL patient, who also carries an autosomal recessive WRN mutation. Although WS carriers are known to be phenotypically normal, we cannot rule out the remote possibility of an oligogenic interaction in which the single recessive *WRN* mutation could somehow modulate the progeroid phenotype. Vigilance for and treatment of complications remain the mainstay of management in both conditions.

The clinical ambiguity between WS and MDPL is not unique to this family. Lessel et al^21^ tested 50 patients with a putative diagnosis of nonclassical WS (ie, progeroid syndrome without WRN, LMNA, BANF1, and ZMPSTE24 mutations) and found causative *POLD1* mutations in 8 patients. As WES and expanded targeted panels see wider usage, many more cases eluding diagnosis may find their answer in *POLD1* mutations.

In summary, we report the 16th molecularly characterized case of de novo MDPL syndrome, co-occurring with autosomal recessive WS within the same nuclear family. The 2 progeroid disorders share common features, including similar body habitus, facial features, and metabolic complications. Absence of cataracts and malignancies, presence of hearing loss and dental crowding, and early age of onset are useful clues to distinguish MDPL from WS. Occurrence of the de novo *POLD1* variant in a nuclear family in which a causative *WRN* variant was already segregating may be entirely coincidental or could imply some nonrandom temporal relationship, for example, related to altered DNA repair mechanisms consequent to the *WRN* variant. WES is an invaluable tool in complex cases whose genotypes identified by targeted sequencing fail to explain the phenotypic segregation. As recognition of MDPL improves, its clinical spectrum and natural history will be better understood.

## References

[1] Shastry S Simha V Godbole K et al A novel syndrome of mandibular hypoplasia, deafness, and progeroid features associated with lipodystrophy, undescended testes, and male hypogonadism. J Clin Endocrinol Metab. 2010;95:E192-E197. 20631028 10.1210/jc.2010-0419 PMC3050107

[2] Weedon MN Ellard S Prindle MJ et al An in-frame deletion at the polymerase active site of POLD1 causes a multisystem disorder with lipodystrophy. Nat Genet. 2013;45:947-950. 23770608 10.1038/ng.2670 PMC3785143

[3] Elouej S Beleza-Meireles A Caswell R et al Exome sequencing reveals a de novo POLD1 mutation causing phenotypic variability in mandibular hypoplasia, deafness, progeroid features, and lipodystrophy syndrome (MDPL). Metabolism. 2017;71:213-225. 28521875 10.1016/j.metabol.2017.03.011

[4] Johansen CT Dubé JB Loyzer MN et al LipidSeq: a next-generation clinical resequencing panel for monogenic dyslipidemias. J Lipid Res. 2014;55:765-772. 24503134 10.1194/jlr.D045963 PMC3966710

[5] Hegele RA Ban MR Cao H McIntyre AD Robinson JF Wang J. Targeted next-generation sequencing in monogenic dyslipidemias. Curr Opin Lipidol. 2015;26:103-113. 25692347 10.1097/MOL.0000000000000163

[6] Dilliott AA Farhan SMK Ghani M et al Targeted next-generation sequencing and bioinformatics pipeline to evaluate genetic determinants of constitutional disease. J Vis Exp. 2018;(134):e57266. 10.3791/57266 PMC5933375 29683450

[7] Kircher M Witten DM Jain P O’Roak BJ Cooper GM Shendure J. A general framework for estimating the relative pathogenicity of human genetic variants. Nat Genet. 2014;46:310-315. 24487276 10.1038/ng.2892 PMC3992975

[8] Sim NL Kumar P Hu J Henikoff S Schneider G Ng PC. SIFT web server: predicting effects of amino acid substitutions on proteins. Nucleic Acids Res. 2012;40:W452-W457. 22689647 10.1093/nar/gks539 PMC3394338

[9] Lopes MC Joyce C Ritchie GR John SL Cunningham F Asimit J et al A combined functional annotation score for non-synonymous variants. Hum Hered. 2012;73:47-51. 22261837 10.1159/000334984 PMC3390741

[10] Lek M Karczewski KJ Minikel EV et al Analysis of protein-coding genetic variation in 60,706 humans. Nature. 2016;536:285-291. 27535533 10.1038/nature19057 PMC5018207

[11] The 1000 Genomes Consortium; Auton A Brooks LD Durbin RM et al A global reference for human genetic variation. Nature. 2015;526:68-74. 26432245 10.1038/nature15393 PMC4750478

[12] Farhan SMK Nixon KCJ Everest M et al Identification of a novel synaptic protein, TMTC3, involved in periventricular nodular heterotopia with intellectual disability and epilepsy. Hum Mol Genet. 2017;26:4278-4289. 28973161 10.1093/hmg/ddx316 PMC5886076

[13] Farhan SM Robinson JF McIntyre AD et al A novel LIPE nonsense mutation found using exome sequencing in siblings with late-onset familial partial lipodystrophy. Can J Cardiol. 2014;30:1649-1654. 25475467 10.1016/j.cjca.2014.09.007

[14] Farhan SM Wang J Robinson JF et al; FORGE Canada Consortium. Old gene, new phenotype: mutations in heparan sulfate synthesis enzyme, EXT2 leads to seizure and developmental disorder, no exostoses. J Med Genet. 2015;52:666-675. 26246518 10.1136/jmedgenet-2015-103279

[15] Pollex RL Hegele RA. Hutchinson-Gilford progeria syndrome. Clin Genet. 2004;66:375-381. 15479179 10.1111/j.1399-0004.2004.00315.x

[16] Garavelli L D’Apice MR Rivieri F et al Mandibuloacral dysplasia type A in childhood. Am J Med Genet A. 2009;149A:2258-2264. 19764019 10.1002/ajmg.a.33005

[17] Barrowman J Wiley PA Hudon-Miller SE Hrycyna CA Michaelis S. Human ZMPSTE24 disease mutations: residual proteolytic activity correlates with disease severity. Hum Mol Genet. 2012;21:4084-4093. 22718200 10.1093/hmg/dds233 PMC3428156

[18] Oshima J Hisama FM. Search and insights into novel genetic alterations leading to classical and atypical Werner syndrome. Gerontology. 2014;60:239-246. 24401204 10.1159/000356030 PMC3997596

[19] Martin GM. The Werner mutation: does it lead to a “public” or “private” mechanism of aging? Mol Med. 1997;3:356-358. 9234240 PMC2230204

[20] Oshima J Martin GM Hisama FM Werner syndrome. In: Adam MP Ardinger HH Pagon RA et al eds. GeneReviews®. Seattle, WA: University of Washington; 1993-2017. https://www.ncbi.nlm.nih.gov/books/NBK1514/. 20301687

[21] Lessel D Hisama FM Szakszon K et al POLD1 germline mutations in patients initially diagnosed with Werner syndrome. Hum Mutat. 2015;36:1070-1079. 26172944 10.1002/humu.22833 PMC4684254

